# Serum fibroblast growth factor 21 levels after out of hospital cardiac arrest are associated with neurological outcome

**DOI:** 10.1038/s41598-020-80086-7

**Published:** 2021-01-12

**Authors:** Pirkka T. Pekkarinen, Markus B. Skrifvars, Ville Lievonen, Pekka Jakkula, Laura Albrecht, Pekka Loisa, Marjaana Tiainen, Ville Pettilä, Matti Reinikainen, Johanna Hästbacka

**Affiliations:** 1 Division of Intensive Care, Department of Anaesthesiology, Intensive Care and Pain Medicine, University of Helsinki and Helsinki University Hospital, PB 340, 00029 Helsinki, Finland; 2 Department of Emergency Care and Services, University of Helsinki and Helsinki University Hospital, Helsinki, Finland; 3 Department of Intensive Care, Päijät-Häme Central Hospital, Lahti, Finland; 4 Department of Neurology, Helsinki University Hospital, Helsinki, Finland; 5 University of Eastern Finland and Kuopio University Hospital, Kuopio, Finland

**Keywords:** Cytokines, Prognostic markers, Ventricular fibrillation

## Abstract

Fibroblast growth factor (FGF) 21 is a marker associated with mitochondrial and cellular stress. Cardiac arrest causes mitochondrial stress, and we tested if FGF 21 would reflect the severity of hypoxia-reperfusion injury after cardiac arrest. We measured serum concentrations of FGF 21 in 112 patients on ICU admission and 24, 48 and 72 h after out-of-hospital cardiac arrest with shockable initial rhythm included in the COMACARE study (NCT02698917). All patients received targeted temperature management for 24 h. We defined 6-month cerebral performance category 1–2 as good and 3–5 as poor neurological outcome. We used samples from 40 non-critically ill emergency room patients as controls. We assessed group differences with the Mann Whitney U test and temporal differences with linear modeling with restricted maximum likelihood estimation. We used multivariate logistic regression to assess the independent predictive value of FGF 21 concentration for neurologic outcome. The median (inter-quartile range, IQR) FGF 21 concentration was 0.25 (0.094–0.91) ng/ml in controls, 0.79 (0.37–1.6) ng/ml in patients at ICU admission (*P* < 0.001 compared to controls) and peaked at 48 h [1.2 (0.46–2.5) ng/ml]. We found no association between arterial blood oxygen partial pressure and FGF 21 concentrations. We observed with linear modeling an effect of sample timepoint (F 5.6, *P* < 0.01), poor neurological outcome (F 6.1, *P* = 0.01), and their interaction (F 3.0, *P* = 0.03), on FGF 21 concentration. In multivariate logistic regression analysis, adjusting for relevant clinical covariates, higher average FGF 21 concentration during the first 72 h was independently associated with poor neurological outcome (odds ratio 1.60, 95% confidence interval 1.10–2.32). We conclude that post cardiac arrest patients experience cellular and mitochondrial stress, reflected as a systemic FGF 21 response. This response is higher with a more severe hypoxic injury but it is not exacerbated by hyperoxia.

## Introduction

Cardiac arrest causes great morbidity, mortality and economic loss^1^. Neurological injury is the most common cause of death during post-resuscitation care^2^ and less than half of those successfully resuscitated survive without neurological sequelae^3^. The cessation of circulation during cardiac arrest causes ischaemia in tissues, disturbing normal cellular function and resulting in cell death. Reperfusion during cardiopulmonary resuscitation and after return of spontaneous circulation (ROSC) leads to generation of reactive oxygen species (ROS) and an inflammatory stress response^4^. The brain is particularly vulnerable to the hypoxia-reperfusion injury in cardiac arrest due to its high metabolic activity and limited energy reserves^5^, but also extracerebral organ failure complicates recovery^6^. Several markers of the magnitude of the hypoxia-reperfusion injury and associated inflammation after ROSC and their association with the severity of multiple organ failure have been investigated^7–9^.

Fibroblast growth factor (FGF) 21, an endocrine hormone which may play a role in the development of reperfusion injury after cardiac arrest, is a member of the large FGF family. FGF 21 is derived mainly from the liver, and plays an important role in energy metabolism and homeostasis^10^. Oxidative and inflammatory stress activate the integrated mitochondrial stress response leading to FGF 21 upregulation in the liver and the pancreas, but also in skeletal muscle and adipose tissue^11,12^. Critical illness may cause mitochondrial dysfunction and, accordingly, elevated FGF 21 levels have been reported in intensive care patients^13^. FGF 21 has been shown to ameliorate hypoxia-induced oxidative stress and inflammation in cerebral microvascular endothelial cells^14^ and to protect against myocardial ischaemia–reperfusion injury in a rat model^15^. Accordingly, due to the pivotal role of mitochondria in oxidative metabolism and oxidative stress we tested the hypothesis that FGF 21 would reflect the severity of hypoxia-reperfusion injury after cardiac arrest. Based on previous reports from animal models of brain ischaemia indicating that hyperoxic reperfusion exacerbates mitochondrial dysfunction^16^ and impairs long-term neurological recovery^17^, we also asked, if post-arrest arterial blood oxygen partial pressure (PaO_2_) has an impact on the FGF 21 response.

## Methods

### Study setting

This was an observational sub-study of the COMACARE study (NCT02698917). The protocol of the study has been reported elsewhere^18,19^. Briefly, we included unconscious, mechanically ventilated adult (age between 18 and 80 years) Out-of-Hospital Cardiac Arrest (OHCA) patients admitted to the participating ICUs with ventricular fibrillation/tachycardia as initial rhythm and ROSC 10–45 min from the onset of cardiac arrest. All study patients received targeted temperature management of 33 or 36 °C for 24 h. COMACARE was a 2^3^ factorial design study, where patients’ treatment targets were randomized to either normoxia [PaO_2_ 10–15 kPa (75–113 mmHg)] or moderate hyperoxia [PaO_2_ 20–25 kPa (150–225 mmHg)], and high or low normal arterial blood carbon dioxide partial pressure (PaCO_2_) and mean arterial blood pressure (MAP). For the purposes of this study we analyzed data on the patients according to the PaO_2_ target groups. The study protocol was approved by The Research Ethics Committee of the Northern Savo Hospital District (295/13.02.00/2015 §53). The study was conducted according to the Declaration of Helsinki. Deferred written informed consent was obtained from the next of kin and additionally from all those patients who regained sufficient neurological function for independent decision-making.

### Laboratory analysis and control samples

We measured serum FGF 21 with a commercially available sandwich ELISA method (BioVendor Brno, Czech Republic). The detection limit reported by the manufacturer is 7 pg/ml and calibration range 30 pg/ml to 1,92 ng/ml. Intra-assay precision is reported as CV = 2.0% and inter-assay precision as CV = 3.3%. We diluted samples as necessary to receive results within the calibration range. We measured serum FGF 21 concentration from samples collected on ICU admission and 24, 48 and 72 h after OHCA from 112 patients enrolled in the COMACARE-trial in the six participating Finnish ICUs. We used samples from 40 age- and gender-matched non-critically ill emergency room patients from the largest participating center as controls. This control group was not included in the COMACARE-trial. The controls were participants of another study (NCT03494790), where blood samples from patients with suspected infection were collected in an emergency department. The study protocol including FGF21 analysis was approved in the ethical board (HUS 1423/2017). The controls were chosen before FGF21 analysis and none of them was diagnosed as having had a severe infection.

### Outcome

Cerebral Performance Category (CPC) at six months after the cardiac arrest, based on patient records and a telephone interview by an experienced neurologist blinded to the intervention groups, was used as the measure of neurological outcome. We defined 6-month CPC 1–2 as good and CPC 3–5 as poor neurological outcome^20^.

### Statistics

We assessed group differences with the Mann Whitney U test and Fisher’s exact test (continuous and categorial variables, respectively). Correlations between variables were assessed with Spearman’s rho. We calculated time-weighted mean PaO_2_ values for timespans from admission to 24 h and from 24 to 48 h of ICU stay by first calculating the mean value for three-hour periods and second the mean of the three-hour means for a 24-h period. We did this to adjust for the altering frequency in blood gas analysis (for example, poor oxygenation leading to increased frequency of sampling during the first hours of treatment).

We analyzed the association between neurological outcome and temporal change in FGF 21 and the association between PaO_2_ intervention group and temporal change in FGF 21 using linear models with alternative covariance structures and restricted maximum likelihood (REML) estimation, a method well suited for analyzing longitudinal data with occasional missing values^21^. The MIXED procedure of the SPSS program was used. We tested three potential covariance matrixes, namely, compound symmetry (CS), first order autoregressive (AR1) and unstructured (UN). We chose unstructured covariance matrix for the final analyses because it provided the lowest Akaike’s information criterion (AIC) in the presented models. We assessed significant effects and interactions with least-significant-difference tests. The residuals followed a normal distribution on visual inspection in the models, confirming that the assumptions were reasonably met. We used *P* < 0.05 as threshold for significance for main effects and interactions and *P* < 0.0125 as threshold for significance for the four predefined pairwise comparisons of the interaction of sampling timepoint by outcome.

Finally, we tested the independent predictive value of FGF 21 for neurological outcome in logistic regression models adjusting for confounding variables. To reduce the dynamics of FGF 21 concentration to a single value to be used in the logistic regression models, we calculated the average value of FGF 21 concentration from the available timepoints between admission and 72 h. We tested the following variables as potential confounders in univariate logistic regression: age, sex, body mass index (BMI), current smoker, bystander initiated resuscitation, time from collapse to the arrival of the first unit, time from collapse to ROSC, APACHE II score (excluding age), serum neuron specific enolase (NSE) measured 48 h after cardiac arrest. Variables with *P* < 0.3 in the univariate analyses (age, bystander initiated resuscitation, time to ROSC, APACHE II score excluding age and 48 h NSE) were selected for the final models. We performed all statistical analyses with the SPSS software (version 24.0, IBM, Armonk, NY, USA). We used GraphPad Prism software, version 8, https://www.graphpad.com to draw the figures.

## Results

Blood samples available for analysis included 111 samples collected at ICU admission [median (IQR) delay from collapse to admission sample was 200 (160–230) min], 111 samples collected at 24 h, 109 samples collected at 48 h and 106 samples collected at 72 h. Thirty days after cardiac arrest, 36 patients were dead and 76 were alive. The most frequent cause of death was hypoxic ischaemic encephalopathy (32 cases). Six months after cardiac arrest 53 patients had CPC class 1 (good cerebral performance), twenty patients had class 2 (moderate cerebral disability), two patients had class 3 (severe cerebral disability), none had class 4 (vegetative state) and 37 had class 5 (death). Median (interquartile range, IQR) age in forty control patients was 62 (53–69) years and 73% of them were male. The baseline characteristics of the study population are presented in Table 1.

**Table 1:** Characteristics of the study patients (N = 112).

	Good outcome N = 73	Poor outcome N = 39	*P*-value	Missing data Good/poor outcome
Age (years)	58 (51–66)	66 (58–75)	< 0.01*	0/0
Sex (male)	84%	80%	0.61	0/0
BMI (kg/m^2^)	26 (24–29)	26 (23–29)	0.86	0/2
Smoker (yes)	33%	41%	0.50	6/7
Bystander initiated resuscitation (yes)	90%	69%	< 0.01*	0/0
Time to first unit (min)	7.0 (6.0–9.0)	7.0 (5.0–10)	0.91	0/0
Time to ROSC^a^ (min)	17 (15–22)	25 (22–32)	< 0.001*	0/0
APACHE II (point)	27 (24–29)	31 (26–35)	< 0.01*	0/0
PaO_2_ group (high)	47%	54%	0.55	0/0
48-h highest PaO_2_ (kPa)	27 (21–32)	29 (22–31)	0.64	0/0
Time from ICU admission to highest PaO_2_ (h)	2.9 (1.9–8.0)	3.7 (1.7–10)	0.60	0/0
NSE 48 h (ng/ml)	17 (13–25)	49 (25–130)	< 0.001*	0/3
**FGF 21 (ng/ml)**				
Admission	0.66 (0.29–1.5)	1.1 (0.50–2.3)	0.04*	1/0
24 h	0.92 (0.40–1.8)	0.84 (0.53–1.7)	0.87	0/1
48 h	1.2 (0.46–2.3)	1.4 (0.43–2.9)	0.46	0/3
72 h	0.57 (0.19–1.8)	1.2 (0.40–2.8)	0.03*	1/5

Continuous variables: median (IQR), Mann Whitney U test; categorical variables: percentage of the whole, Fisher’s Exact test. Good outcome, 6-month Cerebral Performance Categories (CPC) 1–2; Poor outcome, 6-month CPC 3–5.

*Difference between groups is statistically significant at the *P* < 0.05 level.

^a^ROSC between 10–45 min was an inclusion criterion for the study.

**Figure 1:**
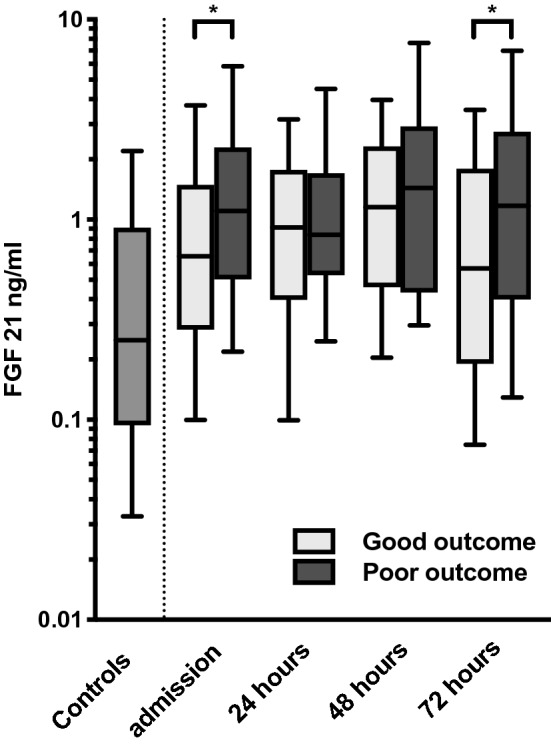
Concentrations of FGF 21 in controls (N = 40) and outcome groups of study patients. Median with interquartile range (box) and 10–90th percentile (whiskers) are presented. Note the logarithmic scale. Good outcome (N = 73), 6-month Cerebral Performance Categories (CPC) 1–2; Poor outcome (N = 39), 6-month CPC 3–5. *Difference between the outcome groups is statistically significant at the *P* < 0.05 level (Mann Whitney U test not adjusted for multiple comparisons).

In the study population the median (IQR) FGF 21 concentration at ICU admission was 0.79 (0.37–1.6) ng/ml and the peak value was 1.2 (0.46–2.5) ng/ml at 48 h. Compared to the ICU admission values, the concentrations in control patients’ samples, 0.25 (0.094–0.91) ng/ml, were significantly lower (*P* < 0.001). The ICU admission FGF 21 concentration was higher in patients with poor neurological outcome [1.1 (0.50–2.3) ng/ml] compared to those with a good neurological outcome [0.66 (0.29–1.5) ng/ml, (*P* = 0.04)]. Linear correlations between admission FGF 21 concentration and patient age, APACHE II score, time from collapse to ROSC or time from collapse to sampling were not observed (supplementary Figs. 1–4). FGF 21 concentrations at the studied timepoints are presented in Table 1 and Fig. 1.

### FGF 21 and oxygen

**Figure 2:**
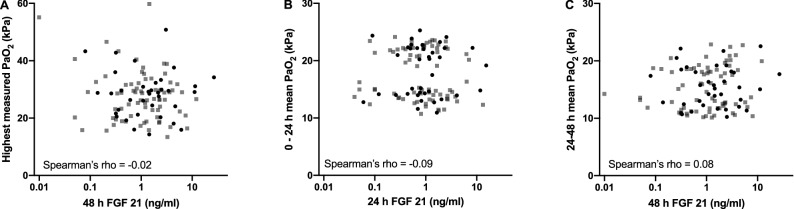
Concentrations of FGF 21 in relation to PaO_2_. (**A**) highest arterial blood PaO_2_ value measured during the first 48 h of ICU stay and FGF 21 concentration measured at 48 h. (**B**) time-weighted mean arterial blood PaO_2_ from admission to 24 h and FGF 21 concentration measured at 24 h. (**C**) time-weighted mean arterial blood PaO_2_ from 24 to 48 h and FGF 21 concentration measured at 48 h. Black circles, poor outcome; grey squares, good outcome.

No significant correlation existed between highest PaO_2_ value during the first 48 h of ICU stay and FGF 21 at 48 h after cardiac arrest (Fig. 2, panel A) or between FGF 21 at 24 h or 48 h and mean PaO_2_ value of the preceding 24-h period (Fig. 2, panel B and C). The distribution of patients with poor and good outcome were comparable over all these scatterplots (Fig. 2).

In the first linear model analysis with REML estimation with FGF 21 concentration as dependent variable, a significant fixed effect of sampling timepoint was found but no effect of PaO_2_ intervention group or the interaction of time by intervention group on FGF 21 concentration (Table 2).

**Table 2:** Effect of timepoint and PaO_2_ group.

Fixed effects on FGF 21 concentration (n = 112)	df	F	*P*-value
Timepoint	3	3.9	0.01*
PaO_2_ group	1	0.09	0.77
Timepoint × PaO_2_ group	3	1.4	0.25

PaO_2_ group, intervention groups targeting normoxia (PaO_2_ 10–15 kPa) or moderate hyperoxia (PaO_2_ 20–25 kPa).

*Statistically significant at the *P* < 0.05 level.

### FGF 21 and neurological outcome

In the second linear model analysis with REML estimation with FGF 21 concentration as dependent variable, there was an effect of sampling timepoint (meaning that FGF 21 concentrations were significantly different between timepoints) and of poor neurological outcome (meaning that FGF 21 concentrations were significantly different between outcome groups) and an interaction of sampling timepoint with poor neurological outcome (meaning that the dynamics of FGF 21 concentration were significantly different between outcome groups) (Table 3). Pairwise comparison of estimated marginal means at different timepoints indicated a significant increase between 24 and 48 h (mean difference 0.83 ng/ml, 95% CI 0.42–1.2 ng/ml, *P* < 0.001, Table 3). The estimated marginal means were higher in the poor outcome group (mean difference 1.2 ng/ml, 95% CI 0.24–2.2 ng/ml, *P* = 0.01, Table 3). Pairwise comparisons of the means defined by sampling timepoint by outcome group interaction, indicated a statistically significant difference at the *P* < 0.0125 level at 72 h timepoint (mean difference 2.5 ng/ml, 95% CI 0.62–4.4 ng/ml, *P* < 0.01, Table 3).

**Table 3:** Effect of timepoint and outcome.

Fixed effects on FGF 21 concentration (n = 112)	df	F	*P*-value
Timepoint	3	5.6	< 0.01*
Poor outcome	1	6.1	0.01*
Timepoint × Poor outcome	3	3.0	0.03*

Pairwise comparisons	Mean difference	95% CI	*P*-value
**Timepoint**			
24 h–admission	− 0.12	− 0.81–0.58	0.74
48 h–24 h	0.83	0.42–1.2	< 0.001*
72 h–48 h	− 0.07	− 0.92–0.79	0.88
**Poor outcome**			
Poor–good outcome	1.2	0.24–2.2	0.01*
**Timepoint × Poor outcome**			
Admission Poor–good outcome	0.68	− 0.58–1.9	0.29
24 h Poor–good outcome	0.36	− 0.61–1.3	0.46
48 h Poor–good outcome	1.4	0.002–2.8	0.05
72 h Poor–good outcome	2.5	0.62–4.4	< 0.01**

Good outcome, 6-month Cerebral Performance Categories (CPC) 1–2; Poor outcome, 6-month CPC 3–5; df, numerator degrees of freedom; CI, confidence interval.

*Statistically significant at the *P* < 0.05 level (Fixed effects and interactions).

**Statistically significant at the *P* < 0.0125 level (four pairwise comparisons of interactions).

In a logistic regression model adjusting for relevant confounding variables, average FGF 21 concentration during the first 72 h after cardiac arrest was independently associated with poor 6-month neurological outcome (CPC 3–5) (Table 4). The results were similar when the logistic regression model was replicated for predicting any 6-month neurological status worse than “good cerebral performance” (i.e. CPC 2–5) (Table 5).

**Table 4:** Logistic regression model for predicting 6-month poor neurological outcome (CPC 3–5).

	OR	95% CI	*P*-value
Age (year)	1.09	1.01–1.17	0.03*
Bystander initiated resuscitation (no)	11.5	1.99–66.5	< 0.01*
Delay to ROSC (min)	1.15	1.04–1.26	< 0.01*
APACHE II (excluding age, point)	0.99	0.84–1.18	0.91
NSE 48 h (ng/ml)	1.11	1.05–1.19	< 0.01*
FGF 21 mean concentration admission to 72 h (ng/ml)	1.60	1.10–2.32	0.01*

Good outcome, 6-month Cerebral Performance Categories (CPC) 1–2 (neurological condition ranging from “good cerebral performance” to “moderate cerebral disability”); Poor outcome, 6-month CPC 3–5 (neurological condition ranging from “severe cerebral disability” to “death”); OR, odds ratio; CI, confidence interval.

*Statistically significant at the *P* < 0.05 level.

**Table 5:** Logistic regression model for predicting 6-month Cerebral Performance Categories 2–5 (neurological condition not classified as CPC 1 “good cerebral performance”).

	OR	95% CI	*P*-value
Age (year)	1.09	1.03–1.16	< 0.01*
Bystander initiated resuscitation (no)	2.31	0.51–10.5	0.28
Delay to ROSC (min)	1.16	1.06–1.26	< 0.01*
APACHE II (excluding age, point)	1.01	0.90–1.14	0.86
NSE 48 h (ng/ml)	1.07	1.01–1.13	0.02*
FGF 21 mean concentration admission to 72 h (ng/ml)	1.50	1.11–2.02	< 0.01*

OR, odds ratio; CI, confidence interval. *Statistically significant at the *P* < 0.05 level.

## Discussion

The main finding of this study is that serum FGF 21 levels were significantly elevated after successful resuscitation in ICU-treated OHCA patients with shockable initial rhythm. Furthermore, the temporal pattern of FGF 21 concentration during the first 72 h was different in patients with poor and good 6-month neurological outcome. We found no association between arterial blood oxygen partial pressures (PaO_2_) and the FGF 21 concentrations in patients after cardiac arrest.

The ischaemia–reperfusion injury associated with cardiac arrest is often followed by an inflammatory reaction resembling septic shock^22^, which may lead to multiple organ failure and death in cardiac arrest patients. In our patient cohort we observed elevated FGF 21 levels on ICU admission and an increase between 24 and 48 h timepoints. Interestingly, FGF 21 has been shown to play an anti-inflammatory role in sepsis^23^ and hypoxia-induced pulmonary hypertension^24^ and to protect myocardium^25^ and neurons^26^ in oxidative stress. Therefore, increasing FGF 21 levels can be interpreted to reflect a protective response to severe perturbations in oxidative metabolism.

Our findings are in line with previous data reporting elevated FGF 21 levels in general population of critical care patients^13^. The emerging role of FGF 21 as a marker of mitochondrial dysfunction^27^ combined with reports of elevated FGF 21 predicting cardiovascular events^28–30^ make cardiac arrest an especially interesting condition for studying FGF 21 due to the extreme ischaemia–reperfusion event of circulatory standstill followed by resuscitation.

Transcription of FGF 21 is regulated by multiple pathways^31^. Disturbance of mitochondrial function leads to upregulation of FGF 21 transcription by ATF4^32^, which is a central player in the integrated stress response signaling network^13,33^. Lactate induces FGF 21 expression in a p38-MAPK dependent manner^34^ and activation of glucocorticoid receptors increases FGF 21 expression^35^. Expression of mRNA in tissues and systemic FGF 21 protein levels change rapidly in response to stressful stimuli. In liver transplants, a robust increase in the expression of FGF 21 in hepatocytes has been reported with peak systemic concentrations in the recipient as early as 2 h after return of hepatic blood flow^36^. In cardiac surgery and ST-elevation myocardial infarction, peak systemic concentrations of FGF 21 were reached 6 h after the insult^37,38^. After a short stressful insult such as cardiac surgery or liver transplantation, systemic FGF 21 returns to basal level in a few days^36,37^, whereas FGF 21 levels in ICU patients remain elevated during the length of the ICU stay^13^.

Based on these data, it is plausible that the peak systemic concentrations of FGF 21 observed at 48 h in the current study are a result of induced transcription in the liver and other tissues in response to hypoxia-reperfusion injury associated with the cardiac arrest. The difference in admission values of FGF 21 between outcome groups (Table 1, Fig. 1) suggests faster elevation in the poor outcome group, although it may also partly reflect the cardiovascular risk factors preceding the cardiac arrest^28–30^. The association of higher FGF 21 concentrations during the first 72 h with poor 6-month outcome (Tables 3, 4, 5) suggest that the stressful process leading to FGF 21 expression fails to resolve in the poor outcome group. In line with this, a downward trend in the median FGF 21 concentration between 48 and 72 h can be seen in the good but not in the poor outcome group (Fig. 1).

Therapeutic hypothermia may have an impact on FGF 21 expression^38^. However, admission samples collected already before cooling had increased FGF 21 levels (Table 1, Fig. 1) and although part of the observed dynamics of FGF 21 after cardiac arrest may be associated with hypothermia, this cannot explain the differences seen between outcome groups (Tables 3, 4, 5) since all study patients received therapeutic hypothermia.

Global tissue hypoxia is considered a central pathophysiological trigger for the post cardiac arrest syndrome^39^. However, after reperfusion of the organs, excess oxygen can also be detrimental. In experimental conditions hyperoxemia during post-resuscitation period has been reported to exacerbate reperfusion injury^16,17^, and retrospective studies of clinical data have suggested that even short exposure to hyperoxia may be associated with mortality in cardiac arrest patients^40,41^. However, our group has been unable to replicate this result in a prospective observational study^42^ and a large registry study^43^. If this association would hold true, mitochondrial injury induced by increased reactive oxygen species production stimulated by hyperoxia would provide a plausible mechanistic explanation. In the current study we found no association between either highest measured PaO_2_ value or preceding 24-h mean PaO_2_ values and FGF 21 concentration as a surrogate marker for mitochondrial stress (Fig. 2).

As discussed above, FGF 21 appears to protect against damaging inflammation, reperfusion injury and oxidative stress when administered to cell cultures or laboratory animals^23–26^. This protective measure can be overwhelmed, and FGF 21 has been shown to predict renal injury after coronary angiography^44^, liver failure in critically ill patients with cirrhosis^45^, and poor outcome in patients with sepsis^46,47^. To the best of our knowledge, there are no previous animal or human studies assessing the FGF 21 response following cardiac arrest or its association with post-resuscitation neurological outcome. In the current study, higher serum FGF 21 concentrations during post-resuscitation care were associated with 6-month poor neurological outcome (Tables 3, 4, 5). The magnitude of the FGF 21 response appears to reflect the severity of the systemic hypoxia-reperfusion injury in cardiac arrest patients, although the large variation in FGF 21 concentrations does not allow its use for prognostication on single patient level.

## Strengths and limitations of the study

This was a multicenter study conducted in a government-funded healthcare system with good research infrastructure. No patient was lost to follow-up and sera were available for all four studied timepoints. The assessor of outcome was blinded and FGF 21 measurements were performed blinded to clinical information. Our study has also limitations. First, the patient population consisted of only OHCA patients with VF/VT as the initial cardiac rhythm and, thus, the generalizability of our findings regarding all cardiac arrest patients is limited. Second, the median time for the first unit to reach the patient was only 7 min, which is not the case in less urban patient cohorts. Third, we had no data on prehospital PaO_2_ or on the pre-arrest FGF 21 levels. Since elevated FGF 21 levels are associated with increased risk of cardiovascular events, there is a possibility that elevated FGF 21 levels after cardiac arrest partly reflect the pre-arrest chronic morbidity of the cardiovascular system and not only conditions associated with the acute event. Finally, this was a rather small pilot study with only 39 poor outcome events, limiting the statistical power of the study.

## Conclusions

Our results suggest a role for FGF 21 in the hypoxia-reperfusion injury following cardiac arrest. Critically ill patients resuscitated after OHCA have elevated FGF 21 concentrations on ICU admission and the temporal pattern and magnitude of FGF 21 response seem to differ according to 6-month neurological outcome. The FGF 21 response is not affected by moderate hyperoxia during ICU care. The large variation on FGF 21 concentrations discourage its clinical use for outcome prediction in cardiac arrest.

## Supplementary Information

Supplementary Information.

## Data Availability

The dataset analyzed during the current study includes sensitive patient information. Legal restrictions prohibit us from making the data publicly available. Data excluding patient identifying information are available from the corresponding author on reasonable request.

## Abbreviations

FGF 21: Fibroblast growth factor 21
REML: Restricted maximum likelihood
