# Supplementary material for: Serum fibroblast growth factor 21 levels after out of hospital cardiac arrest are associated with neurological outcome
Source: Sci Rep. 2021 Jan 12;11:690. doi: 10.1038/s41598-020-80086-7 (PMC7804444; doi:10.1038/s41598-020-80086-7)
Supplement: Supplementary file 1 — Supplementary Information. [file 41598_2020_80086_MOESM1_ESM.pdf]

Serum fibroblast growth factor 21 levels after out of hospital cardiac arrest are associated with neurological outcome

Pirkka T. Pekkarinen[1,*], Markus B. Skrifvars[2], Ville Lievonen[2], Pekka Jakkula[1], Laura Albrecht[2], Pekka Loisa[3], Marjaana Tiainen[4], Ville Pettilä[1], Matti Reinikainen[5], and Johanna Hästbacka[1]

[1]Division of Intensive Care, Department of Anaesthesiology, Intensive Care and Pain Medicine, University of Helsinki and Helsinki University Hospital, Helsinki, Finland
[2]Department of Emergency Care and Services, University of Helsinki and Helsinki University Hospital, Helsinki, Finland
[3]Department of Intensive Care, Päijät-Häme Central Hospital, Lahti, Finland
[4]Department of Neurology, Helsinki University Hospital, Helsinki, Finland
[5]University of Eastern Finland and Kuopio University Hospital, Kuopio, Finland
*Corresponding author

Supplementary figures

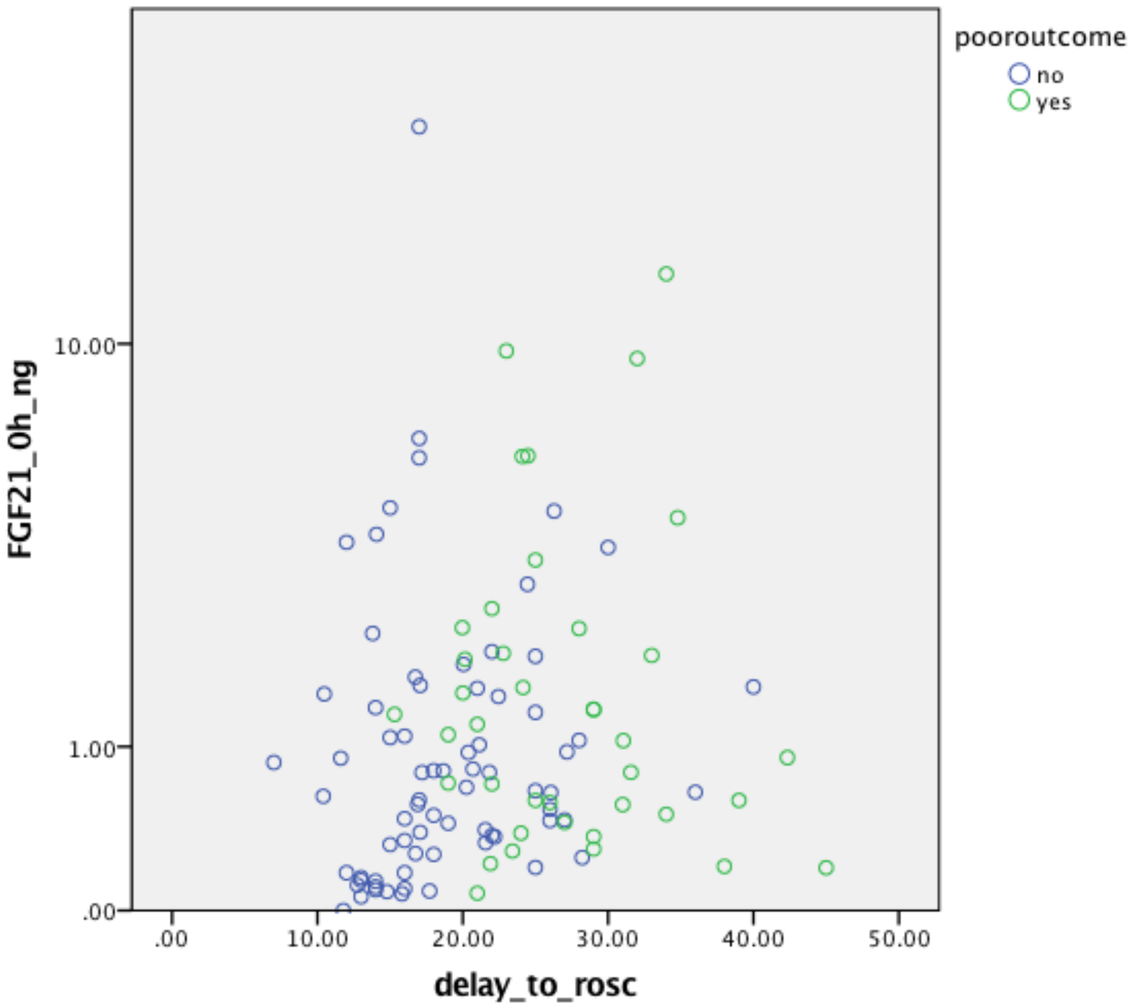

Scatterplot 1: First FGF-21 measurement (admission, ng/ml) plotted against ROSC-delay (min). Blue circles: 6 month CPC 1-2 (good outcome); green circles 6 month CPC 3-5 (poor outcome). Note the logarithmic scale on the Y-axis.

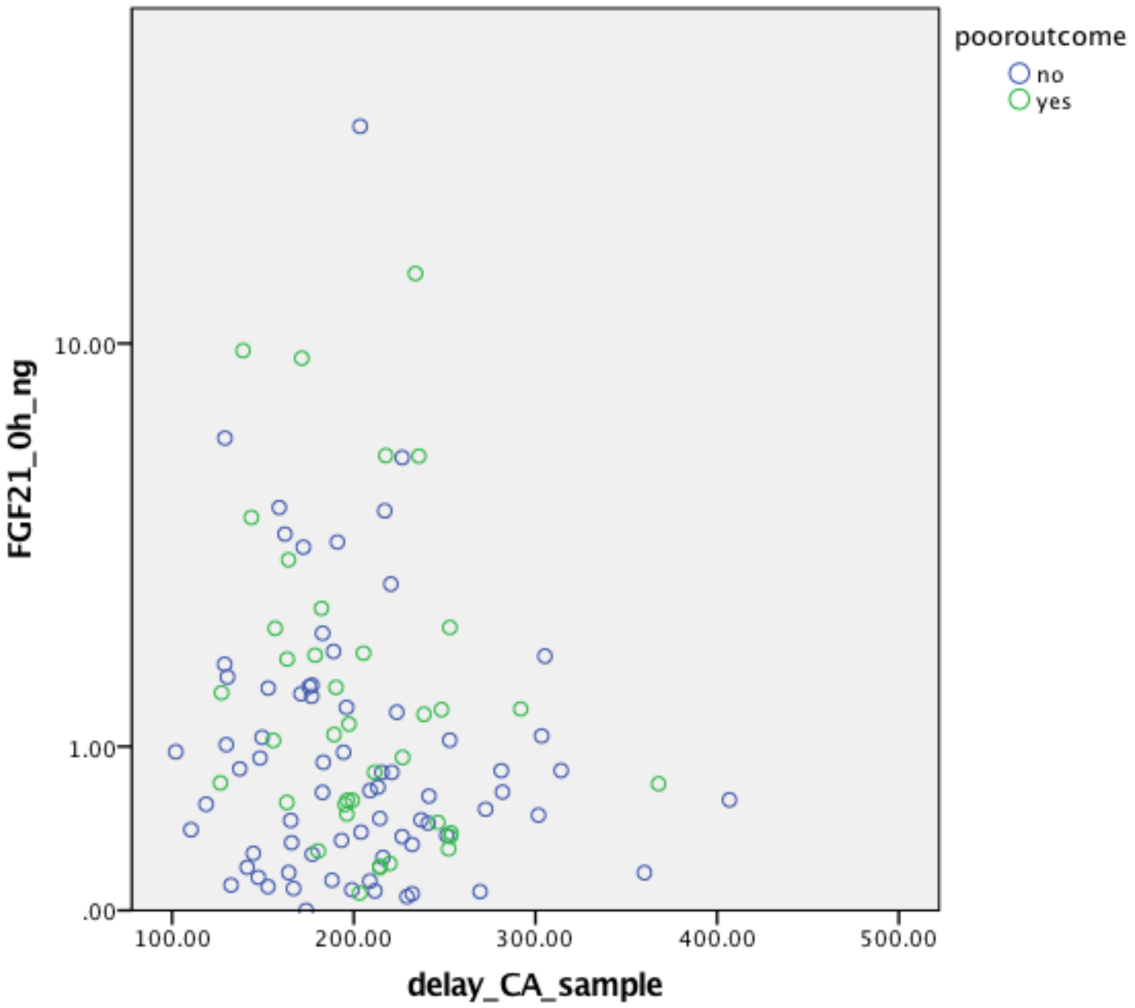

Scatterplot 2. First FGF-21 measurement (admission, ng/ml) plotted against delay from collapse to sampling (min). Blue circles: 6 month CPC 1-2 (good outcome); green circles 6 month CPC 3-5 (poor outcome). Note the logarithmic scale on the Y-axis.

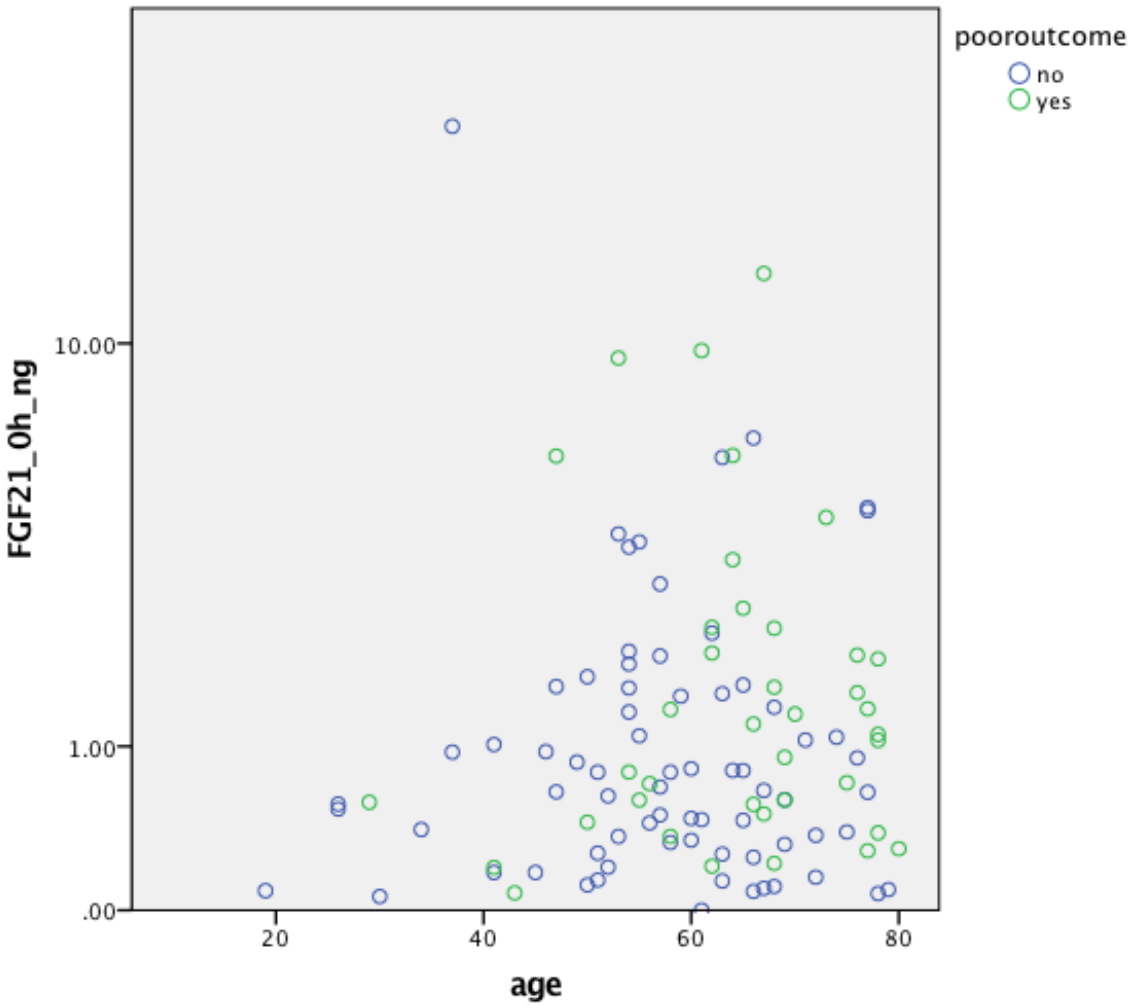

Scatterplot 3. First FGF-21 measurement (admission, ng/ml) plotted against age (years). Blue circles: 6 month CPC 1-2 (good outcome); green circles 6 month CPC 3-5 (poor outcome). Note the logarithmic scale on the Y-axis.

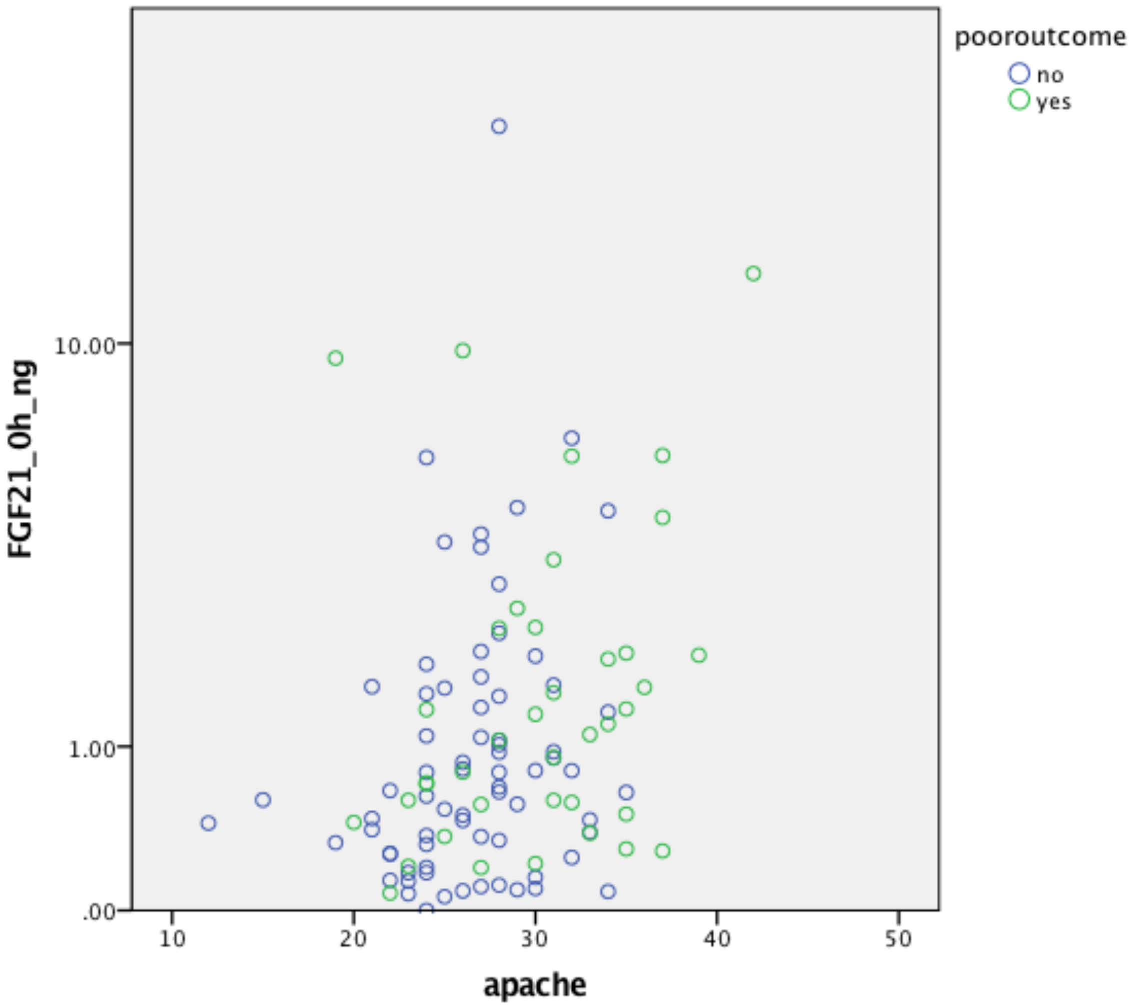

Scatterplot 4. First FGF-21 measurement (admission, ng/ml) plotted against APACHE II score (points). Blue circles: 6 month CPC 1-2 (good outcome); green circles 6 month CPC 3-5 (poor outcome). Note the logarithmic scale on the Y-axis.
